# Anxiety and Depression Symptoms in Spanish Children and Adolescents: An Exploration of Comorbidity from the Network Perspective

**DOI:** 10.1007/s10578-021-01286-4

**Published:** 2021-11-19

**Authors:** Milagros Ocalin Sánchez Hernández, Miguel A. Carrasco, Francisco Pablo Holgado-Tello

**Affiliations:** 1 Department of Psychology, National Autonomous University of Nicaragua, León, Nicaragua; 2 Present Address: Department of Personality Psychology, Evaluation and Psychological Treatment, National University of Distance Education (UNED), Madrid, Spain; 3 Department Methodology of Behavioral Sciences, National University of Distance Education (UNED), Madrid, Spain

**Keywords:** Comorbidity, Depression, Anxiety, Network analysis, Children

## Abstract

The combination of depression and anxiety is among the most prevalent comorbidities of disorders leading to substantial functional impairment in children and adolescents. The network perspective offers a new paradigm for understanding and measuring psychological constructs and their comorbidity. The present study aims to apply network analysis to explore the comorbidity between depression and anxiety symptoms. Specifically, the study examines bridge symptoms, comorbidity, and shortest pathway networks and estimates the impact of the symptoms in the network’s connectivity and structure. The findings show that “feeling lonely” and “feeling unloved” are identified as the most central bridge symptoms. The shortest path network suggests that the role of a mixed anxiety-depressive symptomatology, and specific and non-specific symptoms of clinical criteria, such as “worries,” “feels depressed,” “fears school,” and “talks about suicide” could serve as a warning for comorbidity.

## Introduction

From a standard symptomatological and interdisciplinary perspective, studies in the area of health have confirmed that comorbidity of two or more mental disorders occurs at rates higher than those expected by chance [1–4]. This comorbidity, or joint occurrence, means that an individual is affected by two or more different disorders at the same time. However, comorbidity can be based on different perspectives. Traditionally, there have been two different frameworks: a clinical interpretation framework (i.e., categorical criteria and clinical judgment) and a psychometric or dimensional framework (i.e., assuming the latent variables as proxies of diagnoses). Within a psychometric framework, comorbidity is generally conceptualized as a (bi)directional relationship between two latent variables (i.e., disorders as a cluster of directly related symptoms) that underlie a set of symptoms. More currently, from a network approach [1, 3, 5, 6] focused on individual symptoms and their associations, comorbidity of the two disorders can be explained by specific cross-connections among items from these disorders (i.e., overlapping symptoms/bridge symptoms) instead of either a correlation between two disorders or as the result of a common underlying (neurobiological) dysfunction or “super disorder” [7]. From the network perspective, comorbidity exists when mental disorders have shared symptoms [3, 5, 6], a phenomenon that has become the rule rather than the exception, particularly during childhood and adolescence [1, 8].

The phenomenon of comorbidity is related to an increase in severity, poorer treatment results, increased health system costs, and higher suicide rates [9, 10], underlining the urgent need to study, understand, and address this issue. Depression and anxiety are among the most prevalent comorbid disorders, especially in child and adolescent populations [11–15]; this is alarming because each disorder is independently associated with substantial functional impairment and future mental health problems. Together, they represent a far greater threat to health (e.g., functional impairment, substance abuse, and poorer response to treatment) [16–20].

Although anxiety and depression in youth are meaningfully linked, different theoretical models have proposed important distinctions [11]. According to the *tripartite model*, high physiological arousal is specific to anxiety, while low positive affectivity is specific to depression; however, both share a common component, namely high negative affectivity [21, 22]. The tripartite structure consisting of general distress, physiological hyperarousal (specific anxiety), and anhedonia (specific depression) shares a significant nonspecific component with the anxious and depressed syndromes. This component encompasses general affective distress (negative affect).This tripartite view implies that a complete description of the affective domain requires assessing both the common and the unique elements of the syndromes. Dysfunctional high negative affect essentially signals the presence of either of these disorders (anxiety-depression diagnoses) and differentiation of depression and anxiety is provided by the two specific factors: children who report not only very high levels of general distress, but also both anhedonia and psychophysiological hyperarousal, will be diagnosed as mixed anxiety-depression. However, each disorder will be characterized by general components (conceptualized as negative affectivity or general distress) that contribute to comorbidity among disorders, as well as specific or narrow components that distinguish them (i.e., anxious arousal for anxiety, anhedonia or low positive affectivity for depression). In other words, high negative affect leads to vulnerability of both mood and anxiety disorders, whereas low positive affect is related to depression and high positive affect is related to anxiety.

*Differential emotions theory* postulates that, as an emotion is experienced, it becomes associated with other emotions such that specific emotions tend to occur together or may influence the expression of other emotions. For instance, the emotions of joy, shame, and guilt account for the largest amount of variance in depression scores [23]. From this perspective, disorder comorbidity data between anxiety and depression are likely to reflect shared etiological processes based on a two-factor structure of internalizing disorders: fear disorders (i.e., anxiety disorders or symptoms), and anxious-mood disorders (i.e., depression disorders or symptoms)*.* Thus, the two disorders may have similar emotional features; however, the predominant emotion in anxiety is fear, whereas in depression it is sadness [24–26].

The *multiple pathways model* suggested by Cummings et al. [13] adopts the central proposition of the *tripartite model* distinguishing between fear and distress while also considering heterogeneity among anxiety disorders [13, 27]. Accordingly, some shared and stratified risk factors contribute to the development of the comorbid disorder from multiple potential pathways: (a) youths with a diathesis for anxiety, with subsequent comorbid depression resulting from anxiety-related impairment (Pathway 1); (b) youths with a shared diathesis for anxiety and depression, who may experience both disorders simultaneously (Pathway 2); (c) youths with a diathesis for depression, with subsequent comorbid anxiety resulting from depression-related impairment (Pathway 3). Under this model, anxiety and depression are viewed from both categorical and dimensional perspectives, because examining symptoms or performing diagnosis can lead to different conclusions about the order of onset of anxiety and depressive disorders [13].

On the basis of these models, multiple conceptual explanations could be provided for comorbidity. Among other reasons, disorders could co-occur because they share the same diathesis (i.e., neuroticism, behavioral inhibition), genetic factors, major life events (e.g., loss of a loved one), social-cognitive risk factors, or because one disorder (or some specific symptoms) can trigger the onset of another disorder.

Traditionally, as with many other psychological constructs, the measurement of the relationship between anxiety and depression has been explored methodologically based on the proposition of the *reflective latent variable models*, which consider the items reflecting the manifestation of a latent variable or those reflecting an underlying psychological construct or entity [28, 29]. However, in recent years, the *network perspective* has changed the way psychological constructs are understood and measured. In this perspective, psychological attributes exist as systems whose components are autonomous causal entities that mutually influence each other [3, 29], while highly “central” symptoms (those with stronger inter-symptom connections) spread symptom activation throughout the network [30].

The associations within the system can be examined through two key analytical approaches. One approach involves computing regularized partial correlations networks using a penalized algorithm that ensures only the most significant relationships remain. The other utilizes centrality indices (*strength*, *betweenness*, *closeness*, and *expected influence*) to identify the nodes that have the strongest edges, lead to the shortest distance, and act as the best intermediaries between the connected components [31–33]. The dynamic perspective of this analytical approach makes it possible to identify the *shortest path* between specific nodes in the network by visually highlighting the most significant edges within it [34], and to analyze the *individual node impact* in a network by estimating whether networks could vary in structure and connectivity depending on the levels of symptoms [35].

Comorbidity has been explored using a network perspective in multiple studies [30]. Cramer et al. [2] hypothesize that it arises when there are symptoms (e.g., sleep disturbances and fatigue, concentration problems, and restlessness) bridging two disorders (e.g., depression and generalized anxiety). These are called *bridge symptoms* and can spread activation from one disorder to the other (e.g., worry as core symptom of generalized anxiety leads to sleep problems and fatigue, which leads to a sad mood as a central symptom of depression). They also propose a method for visualizing comorbidity networks in which criteria for key aspects of interpretation about relationships and the positioning of nodes are taken into account. The more two symptoms co-occur, the thicker the edge will look, and overlapping symptoms are placed in the middle of the graph while non-overlapping ones are placed on the extreme left and right.

Boschloo et al. [6], exploring psychological symptoms criteria for diagnosing clinical disorders, conclude that all diagnoses are connected via specific symptom pairs to at least three other diagnoses. Fried et al. [9] indicate that one implication of the network view on comorbidity is that diagnoses may co-occur as a function of their number of shared symptoms. At the time, this was empirically unresolved because, in general terms, the studies measured and visualized the bridge symptoms using traditional network centrality measures [31, 32]. Jones, Ma, and McNally's 2019 study [3] presents formal quantitative methods for identifying bridge symptoms by developing four network statistics, called *bridge centrality measures*, considering the *community* (defined as the theoretically based group of nodes corresponding to a psychiatric disorder based on clinical criteria, not based on any network analytic procedure) [3, 36].

Research on the comorbidity of depression and anxiety symptoms applying network analysis has found that “concentration problems” and “feeling sad” are central symptoms for depression, while a relevant co-occurrence between “loneliness” and other symptoms was found through its association with loss and a lack of instrumental social support even in adulthood [37]. Feelings of restlessness, fatigue, and fear were also found as central symptoms on an internalizing symptom network in a clinical sample of 8–18-years old [38]. Across ages, an increase in connectivity throughout aging development suggests that symptoms may reinforce each other, potentially contributing to the high levels of lifetime continuity for these disorders [39]. Although these are important findings, there is a need for deeper exploration of the comorbidity between depression and anxiety.

The aim of this study is to explore the comorbidity between depression and anxiety symptoms in the context of the knowledge outlined above. The specific aims are (a) to determine the bridge centrality measures for each node and identify bridge symptoms; (b) to explore the associations of the symptoms between the communities by creating a comorbidity network and a shortest pathway network; and (c) to analyze the impact on the strength and structure of the comorbidity network.

Given the high comorbidity that exists between these two disorders, the literature presents us with several expected findings. Regarding the structure and dynamics of the network, we expect from a general view that measures of centrality will indicate: (1) that various symptoms are the most central (hypothesis *a*); and (2) that numerous bridge symptoms can be identified (hypothesis *b*). In accordance with these general results, we expect (3) that the comorbidity network will be highly interconnected and the shortest pathway could vary in relation to the nature of the symptoms (hypothesis *c*); that (4) symptoms related to negative affect may function as bridge symptoms, since this construct has been found to be a component of both depression and anxiety (hypothesis *d*); and (5) interpersonal symptoms will show higher scores on bridge centrality measures given the capacity of interpersonal behaviors to activate other symptoms in the network (hypothesis *e*).

## Methods

### Participants

The sample consisted of 986 Spanish children and adolescents, including 540 girls (55%) and 446 boys (45%). Their ages ranged from 9 to 18 years (*M* = 13.09; *SD* = 2.01). Participants were selected from various public and charter schools in several Spanish cities. As shown in Table 1, the distribution of participants according to age and sex variables was homogeneous (*x*^*2*^ = 2.56; *gl* = 3; *p* = 0.47).

**Table 1:** Distribution of participants by sex and age groups

Sex	9–10 years old	11–12 years old	13–14 years old	15–18 years old	Total
Boys	47	119	167	113	446
Girls	60	157	176	147	540
Total	107	276	343	260	986

### Procedure

The Ethics Commission of the National University of Distance Education (UNED) approved the study and its compliance with the ethical and data protection standards required by European legislation. Approval was then requested from the schools and informed consent was sought from the parents and the participants themselves. Data collection was subsequently carried out in the classrooms with the class groups already established. All the questionnaires were identified using codes to ensure participants’ anonymity. Participation was voluntary and the instructions and evaluation conditions were similar for all participants.

### Measures

Clinical symptoms of depression were measured using the *Center for Epidemiological Studies Depression Scale for Children and Adolescents*, CES-DC [40–43]. This scale groups behaviors into depressed affect, somatic problems, interpersonal problems, and positive affect. It consists of 20 items with four Likert-type response options (from 1 = “almost nothing” to 4 = “a lot”).

The *Youth Self-Report*, YSR [44, 45], was used to study the symptoms of depression-anxiety. The YSR uses self-report of symptoms to evaluate emotional and behavioral problems in children and adolescents. It has 112 items measured on a Likert scale with three answer options (from 0 = “not true” to 3 = “true, very often or fairly often”). The higher the score on the subscales, the higher the degree of psychopathology. The present study only used data from the depression-anxiety subscale, which mixes the manifest behaviors of the two disorders. Two expert clinicians (95% inter-rater reliability) used the Diagnostic and Statistical Manual of Mental Disorders [46] to identify depression and anxiety items by their content (e.g., negative affect, somatic, cognitive, interpersonal) and specificity (i.e., specific versus non-specific). Specific items formed part of the particular or essential criteria for depression or anxiety. Non-specific items, meanwhile, were associated with either anxiety or depression as part of the nomological network of these disorders or were used to operationalize the clinical significant criterion as an additional requirement (e.g., duration, severity, family, social and work/school discomfort/distress or impairment). The items used in this study are shown in Table 2.

**Table 2:** Depression and anxiety-depression symptoms

Long label	Short label	Construct the item measure	Item content notes	Mean	Standard deviation	Median	Skew	Kurtosis	Standard error
Bothered more than usual	CES1	Depression	Irritability/negative affect	1.65	0.77	2	1.16	1.07	0.02
Poor appetite	CES2	Depression	Somatic	1.73	0.86	2	1.09	0.5	0.03
Trouble focusing	CES5	Depression	Somatic	2.12	0.95	2	0.49	−0.68	0.03
Everything has been an effort	CES7	Depression	Somatic	2.37	1	2	0.21	−1.01	0.03
Sleeps restlessly	CES11	Depression	Somatic	1.8	0.96	2	1.03	0.03	0.03
Talks less than usual	CES13^*^	Depression	Related to mutism, social avoidance, withdrawn	1.78	0.89	2	1.03	0.29	0.03
Trouble getting active	CES20	Depression	Somatic/negative affect-anhedonia	1.53	0.82	1	1.56	1.68	0.03
Not able to feel happy	CES3	Depression	Negative affect-anhedonia	1.5	0.82	1	1.67	2.01	0.03
Feels depressed	CES6	Depression	Negative affect-anhedonia	1.77	0.94	1	1.07	0.15	0.03
Thinks life has been a failure	CES9^*^	Depression	Cognitive bias associated with depression but not included in its diagnostic criteria; although it can be interpreted as close to uselessness or devaluation, which is a specific criterion, it is not the same	1.33	0.71	1	2.36	5.14	0.02
Feels fearful	CES10^*^	Depression	Symptom of anxiety	1.48	0.72	1	1.52	1.99	0.02
Feels lonely	CES14^*^	Depression	Interpersonal content of clinical social impairment	1.46	0.8	1	1.78	2.44	0.03
Having crying spells	CES17	Depression	Negative affect-anhedonia	1.83	0.99	2	0.96	−0.21	0.03
Feels sad	CES18	Depression	Negative affect-anhedonia	1.79	0.91	2	1.03	0.2	0.03
Feels just as good as others	CES4	Depression	Interpersonal/positive affect	2.75	1.04	3	−0.33	−1.06	0.03
Feels hopeful	CES8	Depression	Negative affect-anhedonia	2.75	1.02	3	−0.28	−1.07	0.03
Being happy	CES12	Depression	Positive affect	3.37	0.83	4	−1.19	0.59	0.03
Enjoys life	CES16	Depression	Positive affect	3.22	0.9	3	−0.91	−0.15	0.03
People have been unfriendly	CES15^*^	Depression	Interpersonal content of clinical social impairment	1.5	0.8	1	1.63	1.95	0.03
I feel people dislike me	CES19	Depression	Interpersonal/self-esteem	1.56	0.88	1	1.54	1.41	0.03
Fears	YSR29	Anxiety-depression: anxiety	Emotional and phobic content	0.42	0.65	0	1.25	0.33	0.02
Fears school	YSR30	Anxiety-depression: anxiety	Emotional and phobic content	0.06	0.26	0	5.12	27.99	0.01
Fears doing something bad	YSR31	Anxiety-depression: anxiety	Emotional and phobic content/close to obsessive syndrome	0.44	0.64	0	1.13	0.14	0.02
Must be perfect	YSR32^*^	Anxiety-depression: anxiety	Not defining criteria for anxiety; close to obsessive syndrome	0.48	0.66	0	1.05	−0.08	0.02
Feels unloved	YSR33^*^	Anxiety-depression: depression	Not defining criteria for anxiety interpersonal/self-esteem	0.17	0.44	0	2.62	6.32	0.01
Feels worthless	YSR35	Anxiety-depression: depression	Emotional content of low self-esteem	0.28	0.53	0	1.76	2.18	0.02
Nervous/tense	YSR45	Anxiety-depression: anxiety	Physiological content	0.72	0.7	1	0.45	−0.91	0.02
Anxious	YSR50	Anxiety-depression: anxiety	Perceived trait personality/physiological content	0.34	0.55	0	1.41	1.02	0.02
Feels too guilty	YSR52	Anxiety-depression: depression	Emotional content	0.3	0.54	0	1.64	1.75	0.02
Self-conscious	YSR71	Anxiety-depression: anxiety	Cognitive content	0.64	0.72	0	0.67	−0.83	0.02
Talks about suicide	YSR91	Anxiety-depression: depression	Cognitive/interpersonal content	0.06	0.29	0	4.93	25.38	0.01
Worries	YSR112	Anxiety-depression: anxiety	Cognitive content	0.93	0.76	1	0.11	−1.25	0.02

Depression items belong to CES-DC; anxiety-depression items belong to YSR. ^*^Non-specific symptoms of the construct they measure but that are associated with the nomological network of the construct or are used to operationalize the clinical significant criterion as an additional requirement (e.g., duration, severity, family, social and work/school discomfort/distress or impairment)

### Data Analysis Plan

R Program [47] was used to conduct all the analyses. First, an exploratory descriptive analysis of the items was performed. In total, the highest percentage of missing data was 2% for the CES-DC items and 3% for the YSR items. It is standard to consider percentages under 20% to be candidates for imputation [48]. Multiple imputation is recommended as the best method for Likert-type scales [49, 50], even for the CES-DC [51]. Among the multiple imputation techniques used, the random forest approach is considered the most accurate for considering the various patterns of missing data [52–54]. Hence, in the present study, missing data were imputed with multiple imputation via the random forest technique using the MICE package [55].

To explore the comorbidity between symptoms of depression and anxiety-depression from the network perspective, bridge symptoms were determined based on four measures of bridge centrality: the *bridge strength* and *expected influence*, which estimates a node’s sum connectivity with other disorders and differs by taking or not taking the absolute value of edges before summing them; *bridge betweenness*, which assesses the number of times a node lies on the shortest path between any two nodes from two distinct disorders; and *bridge closeness*, which reflects the average distance from a node to all nodes outside of its own disorder. In sum, all are estimated based on the number of edges and edge weights, distance, and intermediation of the nodes in the network [3]. The concordance on bridge symptoms by measures of bridge centrality was visualized using a co-occurrence graph [56].

The comorbidity and shortest pathway networks between disorders were also visualized. The bridge symptoms shown in the first network were determined as top scoring nodes given the bridge centrality measures as it is the suggested method to detect bridge symptoms [3]. The second network can be seen as a roadmap that allows clear identification of possible pathways and mediating items between nodes [34, 57]. Lastly, the impact of each symptom on the global strength and structure of the comorbidity network was studied [35].

Bridge centrality, bridge symptoms, and impact were estimated using the *bridge* and *impact* functions in the *networktools* package [58]. For the comorbidity and the shortest pathway networks the *qgraph* package [34] was also used, with the functions *qgraph* applying method EBICglasso and *pathways*.

## Results

### Bridge Centrality Measures and Bridge Symptoms

**Fig. 1:**
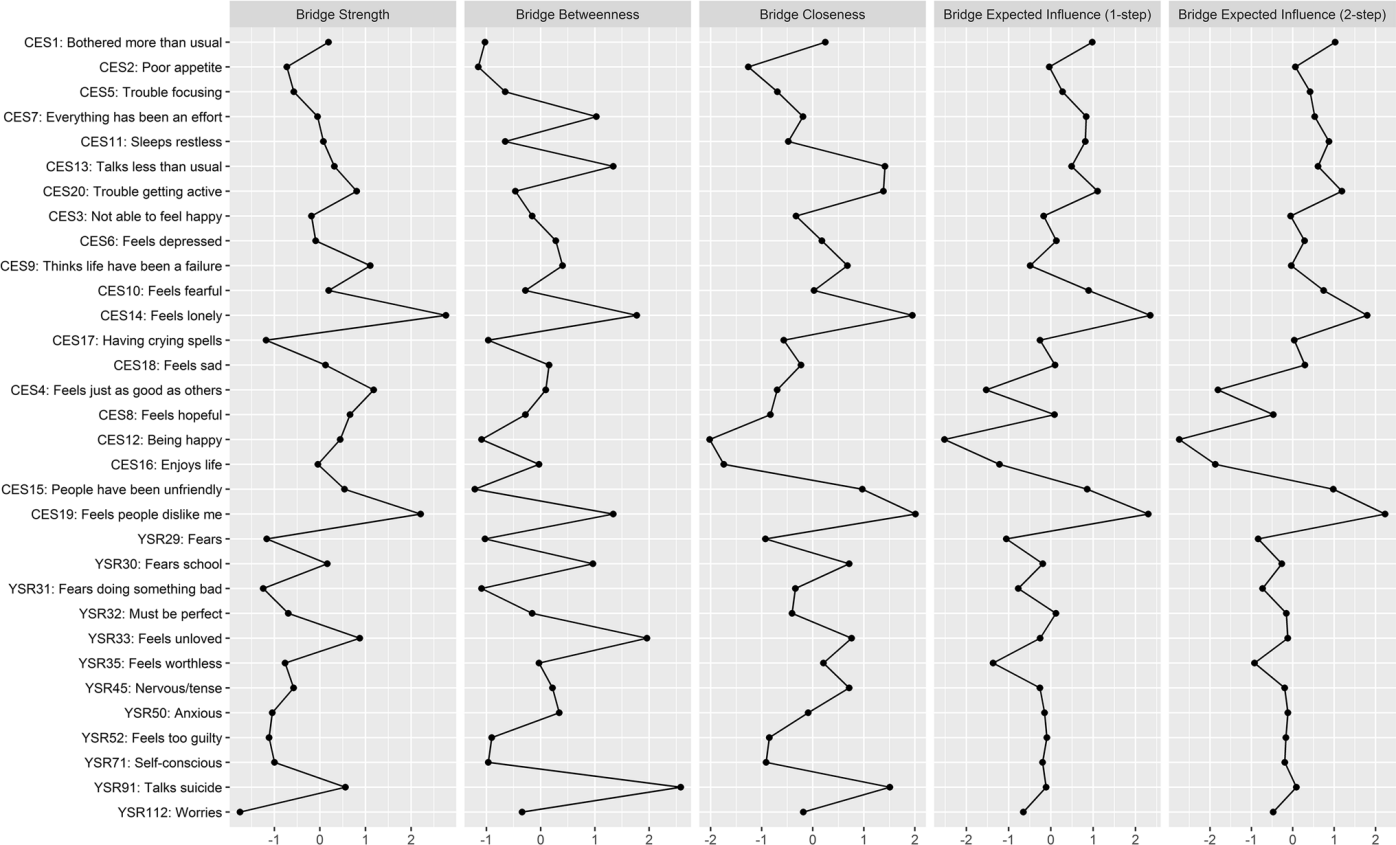
Standard bridge centrality measures of depression and anxiety-depression symptoms in Spanish children and adolescents

Bridge centrality measures were determined for each symptom. The first ones are shown in Fig. 1. The symptoms that are relevant due to their strong connectedness between disorders (bridge strength > 1.00 and expected bridge influence steps 1 and 2 > 1.00) are “feels lonely” (CES14), “I feel people dislike me” (CES19), “trouble getting active” (CES20), “people have been unfriendly” (CES15), and “bothered more than usual” (CES1).

The symptoms that are the greatest intermediaries (bridge betweenness > 1.00) between nodes from both disorders are “talks about suicide” (YSR91), “feels unloved” (YSR33), “fears school” (YSR30), and, in common with the symptoms with the strongest connectedness, “feels lonely” (CES14) and “I feel people dislike me” (CES19).

The symptoms that show the shortest distance (bridge closeness > 1.00) between the two disorders are “feels lonely” (CES14), “I feel people dislike me” (CES19), "trouble getting active” (CES20), “talks about suicide” (YSR91), and “talks less than usual” (CES13).

**Fig. 2:**
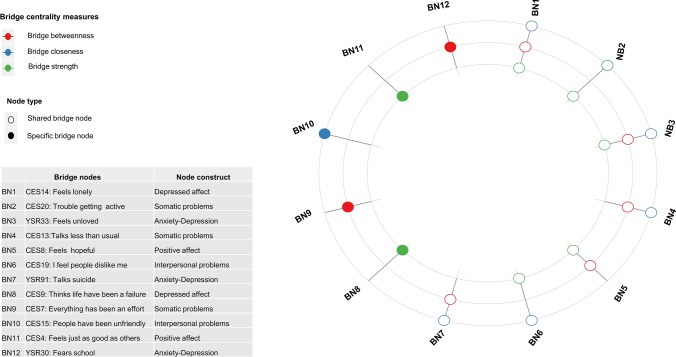
Co-occurrence of bridge nodes according to the measures of bridge centrality of depression and anxiety-depression symptoms in Spanish children and adolescents

Based on the results of the bridge centrality measures, the bridge symptoms of the comorbidity network between anxiety and depression were estimated; these are shown in Fig. 2. The node type differentiates those that are bridge symptoms in more than two measures of bridge centrality (shared bridge nodes), and the specific symptoms are those that have a single measure of bridge centrality (specific bridge nodes).

Both “feels lonely” (CES14) and “feels unloved” (YSR33) were identified as bridge symptoms in all the bridge centrality measures. The combination of bridge strength and bridge closeness further pointed to “trouble getting active” (CES20) and “I feel people dislike me” (CES19); while the combination of bridge betweenness and bridge closeness added “talks about suicide” (YSR91) and “talks less than usual” (CES13). The specific bridge symptoms “thinks life has been a failure” (CES9) and “feels just as good as others” (CES4) were highlighted in bridge strength. “Everything has been an effort” (CES7) and “fears school” (YSR30) were identified in bridge betweenness, and “people has been unfriendly” (CES15) was identified in bridge closeness.

### Comorbidity and Shortest Path Networks

**Fig. 3:**
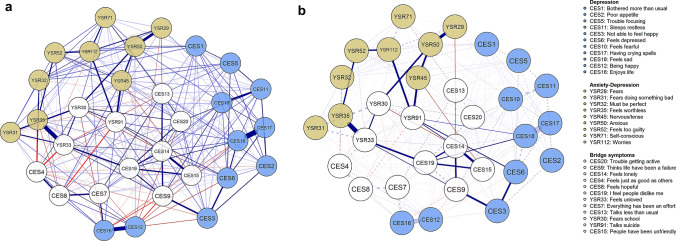
**a** Comorbidity network of depression and anxiety-depression symptoms; **b** Shortest path network between depression and anxiety-depression symptoms in Spanish children and adolescents

To explore the relationships of the symptoms between anxiety and depression, a comorbidity network and a shortest pathway network were created. Both are presented in Fig. 3. In the comorbidity network, moderate and strong correlations (represented by thicker edges) are observed between some nodes of the network. It can be visually identified that the boundaries between the two communities of symptoms are diffuse and clearly interconnected rather than being specific to one or the other disorder.

In Fig. 3b other interesting connections can be observed. The circle from “YSR31: Fears doing something bad” to “YSR30: Fears school” to “YSR33: Feels unloved” and back to YSR31 connects with a symptom of a depressive nature (“YSR35: Feels worthless”). Where “YSR33: Feels unloved” is a symptom apart from anxiety-depression, it can be considered as a warning of comorbidity. Meanwhile, the circle from “CES14: Feels lonely” to “CES15: People have been unfriendly” to “CES19: I feel people dislike me” and back to CES14 connects with suicidal ideation (“YSR91: Talks about suicide”); these items are related to interpersonal relationships.

### Impact of the Symptoms

**Fig. 4:**
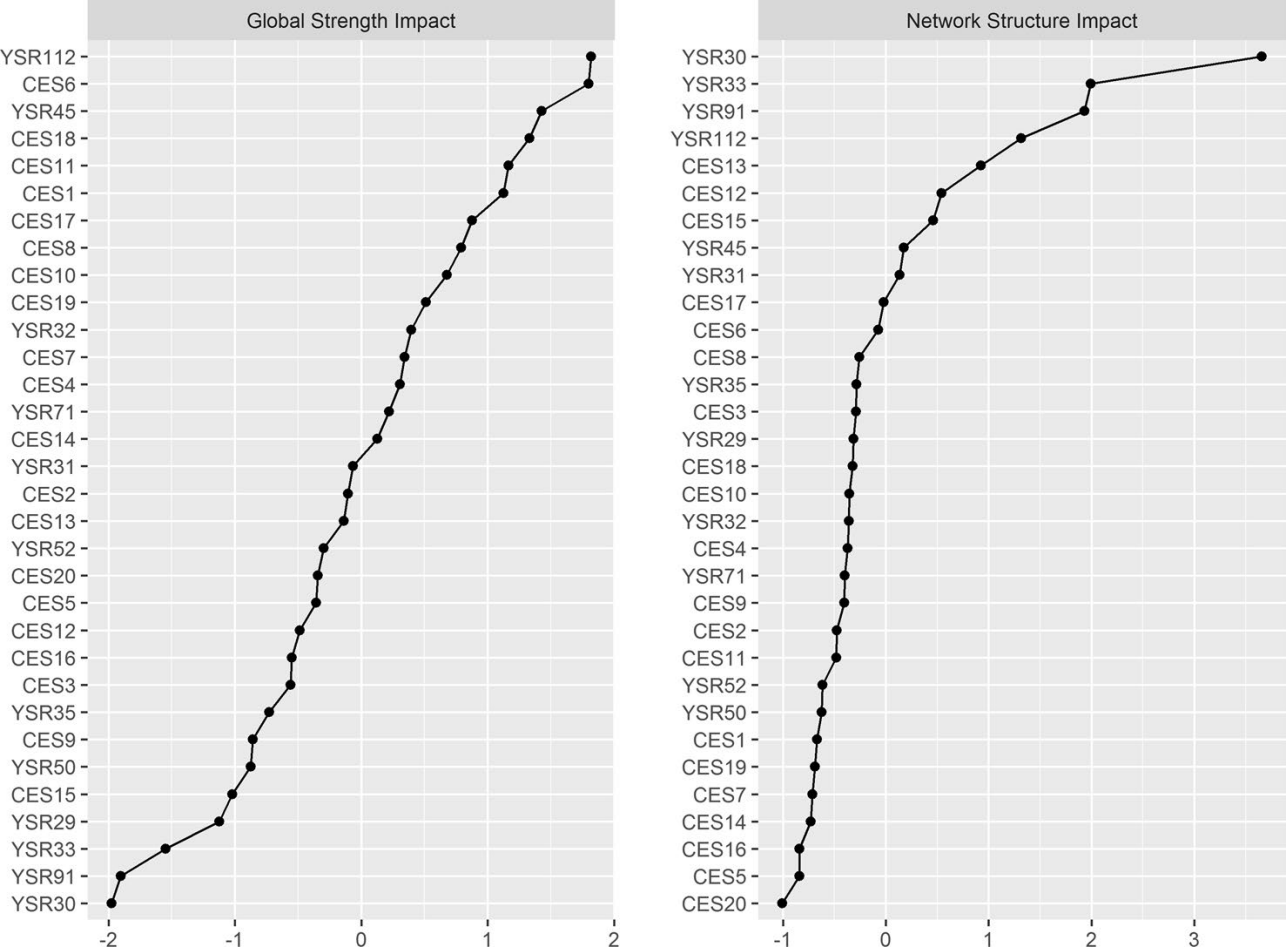
Impact coefficients of the comorbidity network of depression and anxiety-depression symptoms in Spanish children and adolescents

The impact on the strength and structure of the comorbidity network were analyzed. Figure 4 shows that the symptoms with the greatest influence on network connectivity according to the global strength impact coefficient (*GSI* > *1.00*) are “worries” (YSR112), “feels depressed” (CES6), “nervous/tense” (YSR45), “feels sad” (CES18), “sleeps restlessly” (CES11), and “bothered more than usual” (CES1). The symptoms with the greatest potential to cause change in the structure of the network (*NSI* > *1.00*) are “fears school” (YSR30), “feels unloved” (YSR33), “talks about suicide” (YSR91), and “worries” (YSR112).

## Discussion

In exploring the relationships of symptoms of depression and anxiety, it was found through the bridge centrality measures that some symptoms play the role of a bridge system and could thus explain the interconnection or comorbidity between the symptom communities of the respective disorders. This is consistent with hypotheses *a* and *b* and with previous findings [2, 3]. Some of the symptoms involved in the bridge system are non-specific (i.e., “feels lonely,” “people have been unfriendly,” “feels unloved”) and others are specific to anxiety (i.e., “fears school,” “talks less than usual”) or depression (i.e., “bothered more than usual,” “trouble getting active,” “talks about suicide”).

Among those bridge symptoms, all measures of bridge centrality align on “feels lonely” and “feels unloved” as the most significant symptoms in the comorbidity between the disorders. Considering their connotations in relation to the perception and experience of an interpersonal deficit or difficulty (represented by measures such as “people have been unfriendly,” “fears school,” “talks less than usual,” and “I feel people dislike me”), all appear to be associated with psychological maladjustment emerging from several problems whose interactions build a connected network of symptoms. In this sense, the notion of mental health would correspond to a stable state of a weakly connected network of symptoms, while psychological maladjustment would correspond to a stable state of strongly connected symptoms [1, 29, 59]; this coincides with the idea of an essential link and effect with social and academic functioning in both depression and anxiety [13, 15].

Symptoms of depression such as social withdrawal, loss of motivation, sleep disturbance, and reduced energy tend to impact a child’s ability to attend school, particularly absenteeism, unexcused absences/truancy, and school refusal [20]. Likewise, children with anxiety problems may refuse to attend school in order to avoid school-related situations that cause distress or negative affect; or to escape aversive social and/or evaluative situations [60, 61]. Both depressed and anxious children may demonstrate social deficits (e.g., low social skills and social status) and, as a result, do not receive positive social reinforcement; and they have more problems coping with negative life events and high stress. Such symptoms also point to children's negative academic cognitions (i.e., poor beliefs about their important role in academic competence and ability to control academic outcomes) and poor academic performance (14). Consistent with hypothesis *e*, the interpersonal symptoms show higher scores on the bridge centrality measures based on their capacity to activate other symptoms in the network.

The significance of “feels lonely” emphasizes the importance of considering the perception of loneliness, understood as the discrepancy or dissatisfaction between the personal desire for social relationships and the relationships that actually exist, in addition to a feeling of physical or emotional disconnection from others [62]. This is of particular concern because, from a theoretical perspective, this is not considered to be a symptom or standard criterion for clinical diagnosis in categorical diagnostic taxonomies [46], despite multiple studies revealing the relevance of this symptom, including in the Spanish adolescent population [63, 64]. Also “feels unloved” acquires importance if one considers how essential family and interpersonal relationships of acceptance and love are throughout the development of an individual [65–68]. Adults with good mental health are typically those who are able to develop adequate socio-emotional competencies in their relationships with peers and authority figures in their childhood and adolescence. They also experience conflict within the framework of an authoritative and democratic parental style, in which there is a reasonable balance between love and control from parental figures [69, 70].

The comorbidity network shows that despite strong interconnectedness, the communities of depression and anxiety symptoms are diffuse. This is to be expected among children, given findings in the previous literature on the theoretical explanations highlighting the coexistence of anxiety and depression, commonly involving a full spectrum of symptoms, even though both are conceived as single and distinct disorders [8, 13, 15, 71]. It is in line with recent empirical findings for The Hierarchical Taxonomy of Psychopathology (HiTOP), a new diagnostic classification in which the symptoms of both anxiety and depression are considered to form part of a “Distress” subfactor inside the “Internalizing” spectra [72, 73]. Also, as expected (hypothesis *d*), the findings are consistent with the common factor of negative affect between anxiety and depression [21].

The shortest pathway network varies, as expected (hypothesis *c*). When a group of items, or symptoms, such as “fears doing something bad,” “fears school,” and “feels unloved,” connects with a symptom of a depressive nature such as “feels worthless,” the symptom of “feels unloved” should be given special attention when the comorbidity is studied. The same is also true when related items such as “feels lonely,” “people have been unfriendly,” “I feel people dislike me” connects with suicidal ideation in the form of “talks about suicide.” All these items relate to interpersonal relationships and, consistent with hypothesis *e* (loneliness/isolation, unkind people), have an important role in maintaining the symptomatology and activating a potential comorbid depression-anxiety network [19, 20, 74].

It is also important to highlight in this circle the role of item “Talks about suicide.” This is an item related to interpersonal (communication about suicide) and cognitive (ideation of suicide) contents. Suicidal ideation and communication have been mainly associated with depression and especially to depression-anxiety comorbidity as a sign of loneliness grows, which can be severe in young people [19]. From an interpersonal perspective, it is important that research shows significant relationships between different interpersonal factors (e.g., poor social support, relationship quality, peer victimization, social rejection, isolation) and suicide behaviors [75–77] (hypothesis *e*).

In a way, these subpaths or circles are related to previous theoretical findings and provide support for them. Some models suggest that anxiety often temporally precedes depression, but the combination of the two marks a particularly heightened vulnerability and negative prognosis [11, 13]. Others support the conclusion that there is a shared common factor, as in the tripartite model, which includes mixed symptoms called “negative affect” [21]. Still others suggest a multiple pathways model [27], which acknowledges that the comorbidity differs based on the type of anxiety disorder; for example, core risk factors (e.g., genetics) interact with interpersonal risk factors (e.g., loneliness) and cognitive vulnerabilities (e.g., hopelessness), leading to depression among children with social phobia [13, 78, 79].

Symptoms of “worries” and “nervous/tense” (associated with anxiety) as well as “feels depressed” and “feels sad” (associated with depression) stand out in terms of their impact on the connectivity of the network. This is congruent with the main diagnostic criteria of both depression (depressed mood) and anxiety (tension/nervousness) [46, 80] and with existing literature on both disorders across development (ages 5–14), which identify feeling “anxious/fearful” and “unhappy/sad” as the most central symptoms [39]. Again, most of these items are related to the negative affect, in line with the tripartite model. Worry (an essential symptom of *generalized anxiety disorder* [GAD]) is a symptom that, despite being specific to anxiety, is very present in depressive disorders. This is also consistent with the literature that asserts that there is no difference between depression and GAD [81–85].

Symptoms that have strong impact on the structure of the network are more related to comorbidity [35]; “fears school,” “feels unloved,” and “talks about suicide” are the three nodes that could warn of a greater risk of comorbidity and, therefore, of severity and dysfunction. This increases in importance when considering the associations between depression and poor school attendance, particularly absenteeism and unexcused absences/truancy [20], and the fact that adolescents who associate with deviant peers are more likely to report a greater intensity (increased frequency and duration and decreased controllability) of their suicidal ideation [75]. These three symptoms may suggest the level of severity and the clinical significance of these psychological problems.

In summary, the hypotheses were partially confirmed. Of the three bridge centrality measures, only “feels lonely” and “feels unloved” were considered the most central bridge symptoms. The comorbidity network was diffuse and interconnected, consistent with the theoretical proposition of a shared common factor of negative affect between anxiety and depression [21]. Further, the pathway network shows at least two routes for the relationships between the symptoms. Both the connection between symptoms of fears and the self-perception of worth and unloved, and symptoms of interpersonal relationships connecting with suicidal ideation, are highlighted.

Given the previous findings, there are some practical implications to be made. When evaluating evolution or prognosis, clinicians could consider these findings when both disorders are present in patients and provoke symptomology that should be attended to. They may consider the most central and impactful bridge symptoms in the comorbidity network as reference points for diagnosis and clinical assessment and also as targets in prevention practices, counseling, and group/community interventions [86]. The latter should take into account that some symptoms involving negative affect, interpersonal connotations, and cognitive biases can be worked on through training and psychoeducational activities with groups of children and adolescents.

It should be mentioned that this study has a limitation in that we did not consider the more physiological symptoms (e.g., tachycardia, dizziness, shortness of breath) associated with anxiety and/or depression. However, the evidence for physiological symptoms related to depression and anxiety problems is not as strong in child samples as it is in adult samples [87]. Another limitation is that this study was carried out with cross-sectional data in the general population, and thus we could neither study a dynamic sequence nor explore any differences by age groups or sex in clinically referred children and adolescents. As the participants were taken from the general and non-clinical population, they did not share a clinical diagnosis of anxiety and depression.

Future research could carry out comparative research by sex and age groups to identify any differences in the symptomatic dynamics of comorbidity during development and across gender groups and could also explore differences related to cultural variables [38, 39]. It could also adopt multi-method and multi-informant approaches, which could be equivalent to the applied idiographic and nomothetic approaches in comparative research studies with clinical populations. Finally, further research could apply additional analyses, such as personalized networks or time-series networks, complemented with qualitative analysis, and consider cross-cultural, transdisciplinary or international research perspectives [14, 88–93].

## Summary

The aim of this study was to explore the comorbidity between depression and anxiety symptoms from the network perspective. The specific aims were (a) to determine the bridge centrality measures for each node and identify bridge symptoms; (b) to explore the associations of the symptoms between the communities by creating a comorbidity network and shortest pathway network; and (c) to analyze the impact on the strength and structure of the comorbidity network. Data were gathered from Spanish children and adolescents aged 9 to 18 years (*N* = 986). Bridge symptoms were estimated through measures of bridge centrality; comorbidity, shortest pathway networks, and the impact of the symptoms on the networks were explored. Both “feels lonely” and “feels unloved” were identified as the most central bridge symptoms among others of interpersonal connotation and specific diagnostic criteria for each disorder. The shortest path network suggests the role of a mixed anxiety-depressive symptomatology that highlights the connection between symptoms of fears and the self-perception of worth and feeling unloved, and the connection between symptoms of interpersonal relationships and suicidal ideation. Lastly, specific symptoms of both anxiety (e.g., “worries,” “nervous/tense”) and depression (e.g., “feels depressed,” “feels sad”) as well as non-specific symptoms (e.g., “fears school,” “feels unloved,” “talks about suicide”) were shown to strongly impact the connectedness and the structure of the network, which can be considered as a warning of comorbidity.
